# Characterization of H3K4me3 in mouse oocytes at the metaphase II stage

**DOI:** 10.1016/j.jbc.2025.110308

**Published:** 2025-05-29

**Authors:** Atsushi Takasu, Toshiaki Hino, Osamu Takenouchi, Yasuki Miyagawa, Zhihua Liang, Shota Tanaka, Tomoya Mimura, Chisato Ida, Yuki Matsuo, Yuna Lee, Haruka Ikegami, Miho Ohsugi, Shogo Matoba, Atsuo Ogura, Kazuo Yamagata, Kazuya Matsumoto, Tomoya S. Kitajima, Kei Miyamoto

**Affiliations:** 1 Faculty of Biology-Oriented Science and Technology, Kindai University, Wakayama, Japan; 2 Department of Biological Sciences, Asahikawa Medical University, Asahikawa, Japan; 3 Laboratory for Chromosome Segregation, RIKEN Center for Biosystems Dynamics Research (BDR), Kobe, Japan; 4 Laboratory of Animal Reproductive Physiology, Faculty of Agriculture, Kyushu University, Fukuoka, Japan; 5 Department of Biological Sciences, Graduate School of Science, The University of Tokyo, Bunkyo-ku, Tokyo, Japan; 6 Bioresource Engineering Division, RIKEN Bioresource Research Center, Tsukuba, Ibaraki, Japan; 7 Environmental Control Center for Experimental Biology, Kyushu University, Fukuoka, Japan

**Keywords:** chromosome, H3K4me3, mouse, oocyte, preimplantation development

## Abstract

Central functions of histone modifications in germ cell and embryonic development have been documented. Accumulating evidence suggests that oocytes possess unique profiles of histone modifications, among which histone H3 lysine 4 trimethylation (H3K4me3) is broadly spread on the mouse oocyte chromosomes at the metaphase II (MII) stage, unlike later embryonic stages. However, the characteristics and developmental roles of H3K4me3 on MII chromosomes are unclear. Here, we discovered that H3K4me3 was abundantly localized on some of the MII oocyte chromosomes facing the cortical side. Using multicolor FISH and CRISPR–Sirius-based labeling of chromosomes, we revealed that the X chromosome tended to be localized at the cortical side with strong H3K4me3 signals. Anchoring oocyte chromosomes to the cortex may play a role in the asymmetric H3K4me3 distribution. Furthermore, we found that the forced removal of H3K4me3 through the overexpression of a specific lysine demethylase in MII oocytes resulted in abnormal chromosome-spindle structure and impaired preimplantation development after *in vitro* fertilization. These findings highlight the developmental function of H3K4me3 in transcriptionally silent MII oocytes.

Oocytes in fully grown follicles resume meiosis upon the hormonal stimulation and are matured to the metaphase II (MII) stage. MII oocytes are ready for fertilization and following embryonic development. To ensure successful development, oocyte chromosomes need to be precisely segregated upon resumption of meiosis. Failures of this process lead to aneuploidy of resulting embryos, recognized as causes of miscarriage and various genetic disorders in humans (1, 2). Therefore, a number of molecules and mechanisms secure the oocyte chromosomal function. Rec8-mediated chromosome cohesion holds sister chromatids of mouse prophase I oocytes without detectable turnover (3), and weakened chromosome cohesion is a cause of aneuploidy in aged oocytes (4, 5). Actin filaments that colocalize with microtubules during meiosis are crucial for the accurate segregation of chromosomes in oocytes (6). The Arp2/3 complex is localized to the cortex of mouse MII oocytes, forming an actin cap, for subsequent asymmetric division to release small polar bodies (7). Intriguingly, these features are relevant to the oocyte-specific machinery for meiotic division. In addition, many reports suggest that the epigenomic status of an oocyte is unique (8), and its disruption leads to aneuploidy (9). Of note is the distinctive pattern of histone H3 lysine 4 trimethylation (H3K4me3) localization observed on the chromosomes of mouse oocytes.

In eukaryotic somatic cells, H3K4me3 is localized relatively narrowly to the promoter regions of actively transcribing genes. H3K4me3 on transcriptional regulation has been clearly documented using embryonic stem cells (10). However, in oocytes, H3K4me3 exhibits a more widespread distribution that extends further downstream into the gene body, forming what is known as “broad domains” (bdH3K4me3) (11). bdH3K4me3 spans over 10 kb in mouse MII oocytes, and the removal of bdH3K4me3 by lysine demethylases, *Kdm5a* and *Kdm5b,* is essential for zygotic genome activation and early embryogenesis (11). Therefore, the function of bdH3K4me3 is associated with transcriptional regulation in the early embryo. However, bdH3K4me3 is already established in transcriptionally silent oocytes, raising a question about whether oocyte chromosomal H3K4me3, distinct from conventional H3K4me3, is solely related to gene expression regulation in the embryo. Furthermore, when H3K4me3 levels were reduced during oocyte maturation by knocking out *Cxxc1*, a lysine methyltransferase, defective spindle assembly, chromosome misalignment, and metaphase I arrest were observed (12). These results suggest that the role of H3K4me3 in oocytes may extend beyond conventional transcriptional regulation.

In this study, we found that H3K4me3 is abundantly localized at the cortical side of mouse MII-stage oocyte chromosomes, positioned near the plasma membrane. Using combined multicolor FISH and immunofluorescence staining techniques, we discovered the biased localization of H3K4me3 across the chromosomes in MII oocytes. The CRISPR–Sirius-based labeling of chromosomes further showed that X chromosome tended to be localized near the cortex with strong H3K4me3 signals. The forced removal of H3K4me3 by overexpression of a lysine demethylase in MII oocytes resulted in abnormal chromosome-spindle structures and impaired preimplantation development. Our findings thus suggest that enrichment of H3K4me3 in mouse MII oocyte chromosomes is important for subsequent development.

## Results

### Accumulation of H3K4me3 on MII oocyte chromosomes at the cortex side

**Figure 1:**
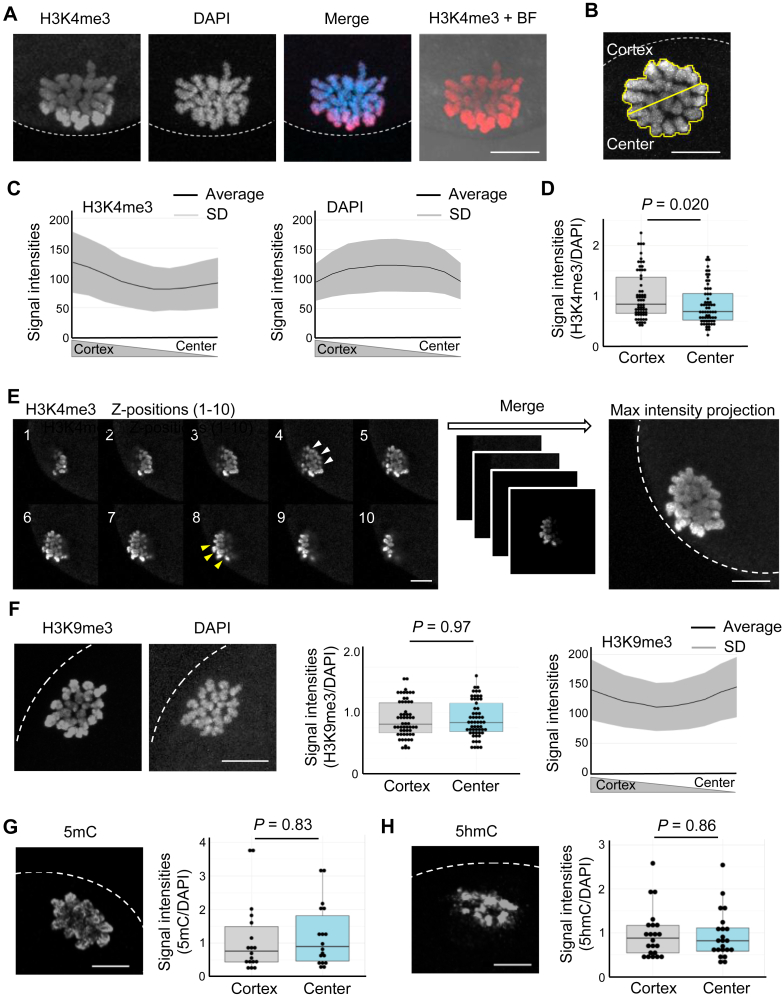
**Enriched accumulation of H3K4me3 on the cortical side of MII oocyte chromosomes.***A*, immunostaining of H3K4me3 using mouse MII oocytes. DNA was stained with DAPI. *Dotted white lines* indicate the cortex of an MII oocyte. BF, bright field; Merge, merged photo. *B*–*D*, quantification of H3K4me3 signal intensities on an MII chromosome. The metaphase plate was selected, and the selected area (*yellow*) was divided into two: the cortex and center sides (*B*). The average signal intensities of H3K4me3 normalized by DAPI signals were compared for each region after subtracting the background signals of H3K4me3 and DAPI, and a box plot was created (*D*). The *line graph* shows the average signal intensities of H3K4me3 or DAPI from the cortex to the center, with the colored area indicating the SD (*C*). N = 9 independent experiments, n = 63 oocytes. *E*, immunostaining images of H3K4me3 across z-stacks using an MII oocyte. All focal planes were merged by maximum intensity projection. *F*, immunostaining of H3K9me3 using MII oocytes and its quantification, as performed in (*B*–*D*). N = 6 independent experiments, n = 57 oocytes. *G*, a representative immunofluorescent image of 5mC using MII oocytes and its quantification. N = 4 independent experiments, n = 18 oocytes. *H*, a representative immunofluorescent image of 5-hydroxymethylcytosine (5hmC) using MII oocytes and its quantification. N = 4 independent experiments, n = 22 oocytes. *p* Values were determined by *t* test. Scale bars represent 10 μm. DAPI, 4′,6-diamidino-2-phenylindole; H3K4me3, histone H3 lysine 4 trimethylation; 5mC, 5-methylcytosine; MII, metaphase II.

Immunofluorescence analyses using an antibody against H3K4me3 (active motif; catalog no.: 39159) revealed uneven accumulation of H3K4me3 on mouse oocyte chromosomes at the MII stage (Fig. 1*A*). To quantify the signal intensities of H3K4me3, MII chromosomes were compartmentalized into two regions, cortex and center sides as depicted in Figure 1*B*. Significant accumulation of H3K4me3 on oocyte chromosomes at the cortex side was observed, whereas 4′,6-diamidino-2-phenylindole (DAPI) signals did not show the cortical localization (Fig. 1, *C* and *D*). The cortically biased pattern of H3K4me3 staining in MII oocytes was also confirmed using another antibody against H3K4me3 (Abcam; ab8580) (Fig. S1*A*). The cortically localized H3K4me3 on MII oocyte chromosomes was seen across the *z*-axis (Fig. 1*E*). Furthermore, the accumulation of H3K4me3 on MII chromosomes adjacent to the cortex was confirmed by the costaining with the cortical actin layer (Fig. S1*B*). These images were obtained using the staining method for chromosomal proteins of oocytes and early embryos, in which Triton X-100 was included during fixation to wash out nonchromatin proteins (13). To rule out the possible artifact of the immunostaining method, oocytes were stained using the widely used staining method; fixation without Triton X-100, followed by permeabilization. This method also resulted in the detection of the cortical accumulation of H3K4me3 on oocyte chromosomes (Fig. S1*C*). We then examined the localization of histones H3K9me3 (Fig. 1*F*), H3K36me3 (Fig. S1*D*), H3.3 (Fig. S1, *E* and *F*), 5-methylcytosine, (Fig. 1*G*), and 5-hydroxymethylcytosine (Fig. 1*H*) on MII oocyte chromosomes, but none of them showed the cortical localization. These results indicate that H3K4me3 is abundantly accumulated in oocyte chromosomes near the cortex.

### Specific chromosomes are cortically localized and marked by abundant H3K4me3

**Figure 2:**
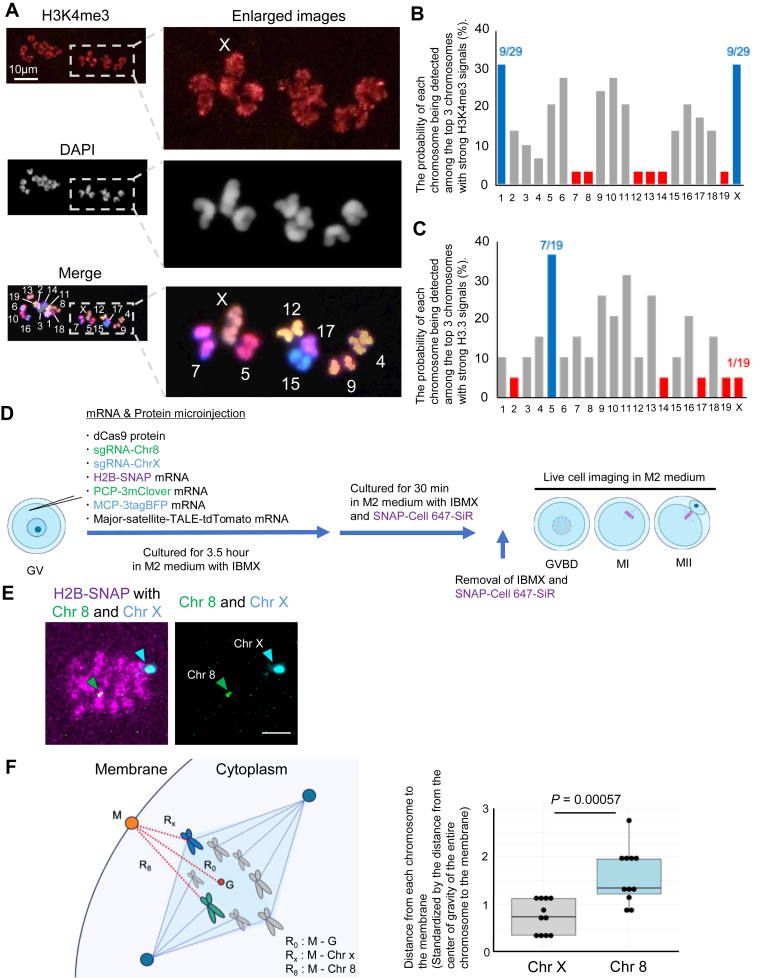
**H3K4me3 is enriched in a set of specific meiotic chromosomes.***A*, multicolor FISH and H3K4me3 staining of MII oocytes. After immunofluorescent staining of mouse MII oocyte chromosomes with H3K4me3, the same samples were subjected to multicolor FISH as depicted in Figure S2*C*. Numbers indicated in the *bottom* merged image correspond to the chromosome numbers. *Dotted squares* in the *left images* are enlarged at the *right panels*. N = 4 independent experiments, n = 29 oocytes. *B* and *C*, the graphs summarize the frequencies (%) of each chromosome to be detected as the top three chromosomes with the strongest H3K4me3 (*B*) or H3.3 (*C*) signals. Each number *above the bar* shows the actual number detected as being strong in H3K4me3 or H3.3 signals among all trials. The *horizontal axis* shows chromosome numbers. *D*, scheme for live cell imaging experiments to observe chromosomes. *E*, visualization of target chromosomes in mouse MII oocytes. Confocal z-sections of H2B-SNAP (chromosomes, *magenta*), MCP-3tagBFP (chromosome X, *cyan*, *cyan arrowhead*), and PCP-3mClover (chromosome 8, *green*, *green arrowhead*) are shown. *F*, distance from each chromosome to the cortex in mouse MII oocytes. The distances from the center of the entire chromosomes to the neighboring cell membrane were designated as R_0_, and those from chromosomes 8 and X to the cell membrane were referred to as R_8_ and R_X_, respectively. These distances were standardized by R_0_. G: center of gravity; M, membrane coordinate. N = 3 independent experiments, n = 11 oocytes. *p* Value was determined by *t* test. Scale bars represent 10 μm (*A*) and 5 μm (*E*). H3K4me3, histone H3 lysine 4 trimethylation; MII, metaphase II.

We asked if the cortical H3K4me3 accumulation was observed in a set of specific chromosomes. The 3D construction of immunofluorescence images of MII oocytes suggests that H3K4me3 may be enriched in specific chromosomes near the cortex (Fig. S2, *A* and *B* and Video S1). Then, the recently reported karyotyping method for mouse oocytes using multicolor FISH (14) was adapted to immunofluorescent analyses (Fig. S2, *C* and *D*, see Supporting *Experimental Procedures* section), allowing the comparison of H3K4me3 signals among different chromosomes. This method can detect H3K4me3 signals on each chromosome (Fig. 2*A*). When the probability of how often each chromosome was recognized as the top three–strongest H3K4me3 signals was calculated, chromosomes 1 and X most often showed strong H3K4me3 signals (Fig. 2*B*; 9 among 29 oocytes). Smaller chromosomes such as chromosomes 12, 13, 14, and 19 were prone to show weaker H3K4me3 levels (Fig. 2*B*). The same analysis was performed against H3.3, and chromosomes 1 and X were not selected as top three–strongest signals unlike H3K4me3 (Fig. 2*C*).

Recently, CRISPR–Sirius-based labeling of each oocyte chromosome has been reported (15). Here, we applied this live cell labeling method to examine whether the X chromosome tends to be located on the cortex side. Initially, single guide RNAs for chromosome X and chromosome 8, as a control, were injected into MII oocytes to trace the kinetics of each chromosome. Still, reliable labeling was hampered probably because of the condensed state of MII chromosomes. Therefore, we injected single guide RNAs for labeling chromosomes X and 8 with different colors and mRNAs encoding the chromosome marker H2B-SNAP and the kinetochore marker Major-satellite-TALE-tdTomato to oocytes at prophase I (Fig. 2*D*). These injected oocytes were *in vitro* matured to the MII stage, allowing us to identify the locations of chromosomes 8 and X in MII oocytes (Fig. 2*E*). The live imaging data revealed the preferential localization of chromosome X on the cortex side in MII oocytes (Fig. 2*F*). Taken together, chromosome X in mouse MII oocytes is often located near the cortex, where H3K4me3 enrichment is observed.

### H3K4me3 on mouse MII oocyte chromosomes is important for subsequent embryonic development

**Figure 3:**
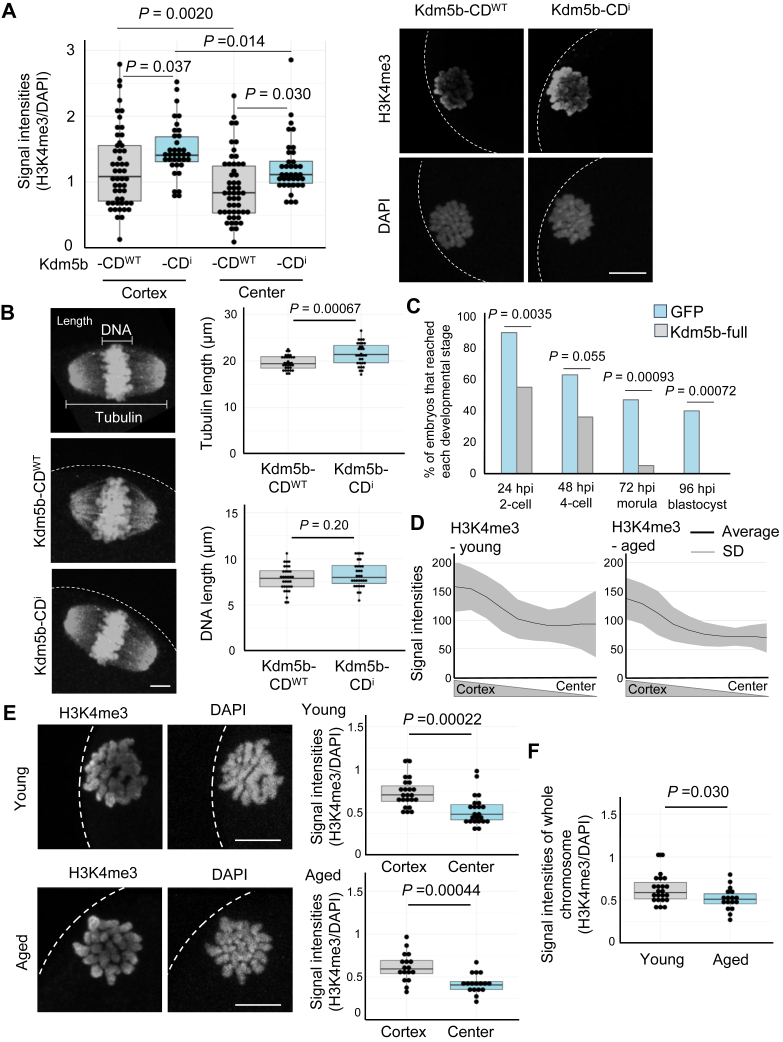
**H3K4me3 levels in MII oocyte chromosomes and their relationship to the spindle apparatus, preimplantation development, and aging.***A*, reduced H3K4me3 signals both at the cortex and center sides by overexpressing *Kdm5b-CD*^*WT*^ in MII oocytes. As a control, catalytically inactive *Kdm5b-CD*^*i*^ was overexpressed. *Box plots* show the quantification of H3K4me3/DAPI signal intensities. Representative images are shown on the *right panels*. N = 3 independent experiments, n = 51 (*Kdm5b-CD*^*WT*^) and 37 (*Kdm5b-CD*^*i*^). *B*, tubulin and DNA sizes of MII oocyte chromosomes overexpressing *Kdm5b-CD*^*WT*^ mRNA, as compared with those overexpressing *Kdm5b-CD*^*i*^. Immunostaining of tubulin in mouse MII oocytes was performed, and the length of tubulin and DNA was measured as explained in the *top panel*. The same images were shown in the *top* and *bottom panels*. *Box plots* show the quantification of each parameter. N = 3 independent experiments, n = 32 (*Kdm5b-CD*^*WT*^) and 31 (*Kdm5b-CD*^*i*^). *C*, impaired preimplantation development after IVF of *Kdm5b*-overexpressed oocytes. Development to each developmental stage was observed every 24 h postinsemination (hpi). As a control, *GFP* mRNA was injected into oocytes. *p* Values were determined by Tukey’s test (*A*), *t* test (*B*), and Chi-squared test (*C*). ∗*p* < 0.05. Scale bar represents 10 μm (*A*) and 5 μm (*B*). *D*, quantification of H3K4me3 signal intensities on MII oocyte chromosomes derived from young and aged mice as performed in Figure 1. The *line graphs* show the average signal intensities of H3K4me3 from the cortex to the center, with the colored area indicating the SD. N = 3 independent experiments, n = 23 (young), 17 (aged) oocytes. *E*, representative images of H3K4me3 immunostaining using MII oocytes derived from young and aged mice. The average signal intensities of H3K4me3 normalized by DAPI signals were compared for each region after subtracting the background signals, and *box plots* were created (*right panels*). DNA was stained with DAPI. *Dotted white lines* indicate the cortex of an MII oocyte. N = 3 independent experiments, n = 23 (young), 17 (aged) oocyte. *F*, H3K4me3 levels in aged oocytes are lower than those in young oocytes. N = 3 independent experiments, n = 23 (young), 17 (aged) oocyte. *p* Values were determined by *t* test. Scale bars represent 10 μm. DAPI, 4′,6-diamidino-2-phenylindole; H3K4me3, histone H3 lysine 4 trimethylation; IVF, in vitro fertilization; *Kdm5b-CDi*, the inactive catalytic domain of lysine demethylase 5B; *Kdm5b-CDWT*, the wildtype catalytic domain of lysine demethylase 5B; MII, metaphase II.

We next examined the developmental roles of H3K4me3 on MII oocyte chromosomes. The catalytic domain of Kdm5b, H3K4-specific demethylase, was exogenously expressed in MII oocytes (Kdm5b-CD^WT^). The expression of Kdm5b-CD^WT^ lowered chromosomal H3K4me3 both at the cortex and central sides when compared with oocytes expressed with Kdm5b-CD^i^, the catalytic inactive version of the enzyme (16) (Fig. 3*A*). The expression of Kdm5b-CD^i^ did not affect H3K4me3 signal intensities and the localization pattern of H3K4me3 in MII oocytes when compared with the noninjected control (Fig. S3, *A* and *B*). After lowering the H3K4me3 levels on oocyte chromosomes by Kdm5b-CD^WT^ expression, the length of the spindle apparatus became shortened as measured by the tubulin staining (Fig. 3*B*). As a control, the expression of Kdm5b-CD^i^ did not alter spindle morphology, when compared with noninjection control (Fig. S3*C*). The oocytes expressed with Kdm5b-CD^WT^ were *in vitro* fertilized, and preimplantation development was observed, but they developed to the blastocyst stage with similar efficiency to control Kdm5b-CD^i^-expressed oocytes (Fig. S3*D*). Then, we expressed the full length of Kdm5b to achieve an efficient reduction of H3K4me3 from oocyte chromosomes. The expression of full Kdm5b achieved 48% reduction of H3K4me3 levels (full Kdm5b *versus* control GFP injection), whereas Kdm5b-CD^WT^ resulted in 22% reduction (Kdm5b-CD^WT^ *versus* control Kdm5b-CD^i^ injection) (Fig. S3*E*). After the expression of full Kdm5b, shortening of the spindle apparatus was also observed (Fig. S3*F*). The Kdm5b-expressed oocytes showed developmental arrest at preimplantation stages after *in vitro* fertilization (IVF) (Fig. 3*C*). These results suggest that H3K4me3 deposited in oocyte chromosomes is important for subsequent development.

### H3K4me3 on MII chromosomes in aged mouse oocytes

Previous studies suggest that some of the histone modifications are abnormally deposited in aged oocytes (17), and altered H3K4me3 in aged mouse oocytes at the germinal vesicle and MII stages was reported (18, 19). We therefore compared the level and pattern of oocyte chromosomal H3K4me3 between young and aged (48 weeks) oocytes. The cortical enrichment of H3K4me3 was observed both in young and aged oocytes (Fig. 3, *D* and *E*). On a global level, oocyte chromosomes from aged mice showed significantly weaker H3K4me3 signals than those from young mice (Fig. 3*F*). These results suggest that the cortical localization is not changed in oocytes collected from aged mice.

### Anchoring the meiotic apparatus to the cortical layer may affect asymmetric H3K4me3 localization

**Figure 4:**
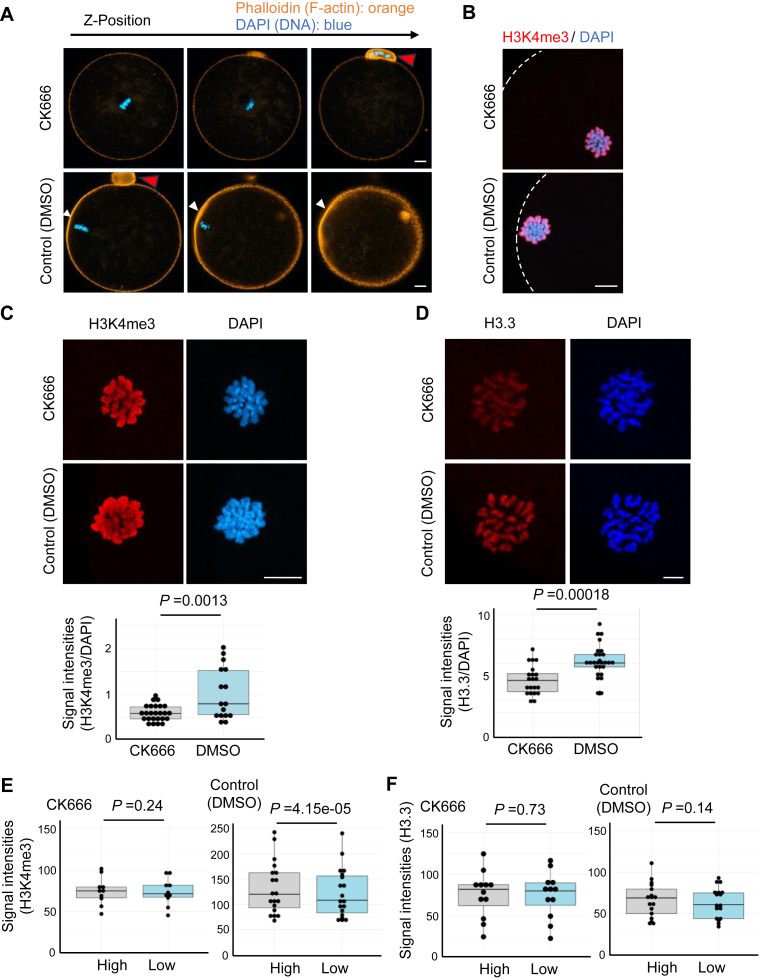
**The dislocation of the meiotic chromosomes from the cortex affects H3K4me3 localization.***A*, the inhibition of Arp2–3 kinetics by CK666 impaired the actin cap formation in MII oocytes. The actin cap structure (*white arrowheads*) was visualized by phalloidin staining (*orange*). DNA was stained by DAPI (*blue*). Images across different z-stacks are shown. Polar bodies are pointed by *red arrowheads*. N = 2 independent experiments, n = 24 (CK666-treated), 18 (control) oocytes. *B*, the inhibition of Arp2–3 kinetics resulted in the dislocation of meiotic chromosomes from the oocyte cortex toward the center of an oocyte. The *blue color* shows DNA stained by DAPI, and the *red color* shows H3K4me3. *Dotted white lines* indicate the cortex of MII oocytes. *C* and *D*, quantification of H3K4me3 (*C*) and H3.3 levels (*D*) in CK666-treated and DMSO-treated (control) oocytes. Representative images are shown above. *Box plots* show the quantification of H3K4me3/DAPI (*C*) and H3.3/DAPI (*D*) signal intensities. N = 3 independent experiments, n = 10 (H3K4me3, CK666-treated), 19 (H3K4me3, control), 12 (H3.3, CK666-treated), and 17 (H3.3, control) oocytes. *p* Values were determined by *t* test. Scale bars represent 10 μm. *E* and *F*, the biased localization of H3K4me3 (*E*) and H3.3 (*F*) signals across MII oocyte chromosomes was analyzed following the method shown in Figure S4. *Box plots* show the quantification of H3K4me (*E*) and H3.3 (*F*) signal intensities in CK666-treated and control oocytes. N = 3 independent experiments, n = 10 (H3K4me3, CK666-treated), 19 (H3K4me3, control), 12 (H3.3, CK666-treated), 17 (H3.3, control) oocytes. *p* Values were determined by *t* test. DAPI, 4′,6-diamidino-2-phenylindole; DMSO, dimethyl sulfoxide; H3K4me3, histone H3 lysine 4 trimethylation; MII, metaphase II.

The cortically located oocyte chromosomes are closely positioned to the actin cap structure, characterized by the localization of actin nucleators and actin filaments (20, 21), and the disruption of the actin cap structure with CK666, a specific inhibitor for Arp2/3, results in the centrally located spindles (22). We examined the effect of the cortically located meiotic apparatus on the asymmetric H3K4me3 localization. The disruption of actin cap by CK666 treatment was confirmed by phalloidin staining (Fig. 4*A*). After CK666 treatment, MII oocyte chromosomes were dislocated from the cortex and moved toward the center of the oocytes (Fig. 4*B*). After the dislocation by adding CK666, the levels of chromosomal H3K4me3 were significantly decreased (Fig. 4*C*). However, control H3.3 staining similarly showed reduced signals when chromosomes moved inward (Fig. 4*D*), possibly because of the placement effect on immunostaining. Thus, the comparison of signal intensities between the treatments was inappropriate in this experimental setting, and we then assessed the asymmetric distribution pattern of H3K4me3 on oocyte chromosomes by dividing chromosomal areas (Fig. S4). These analyses suggested uneven distribution of H3K4me3 signals in control cortically localized chromosomes (Fig. 4*E*, high *versus* low in control oocytes). However, this asymmetric H3K4me3 distribution was lost in the centrally located meiotic apparatus (Fig. 4*E*, CK666). In contrast, H3.3 showed asymmetric distribution in neither CK666-treated nor control oocytes (Fig. 4*F*). Taken together, these results suggest that the cortical localization of oocyte meiotic chromosomes may affect asymmetric H3K4me3.

## Discussion

Unique epigenetic features of oocytes have been revealed (23), among which dynamic changes of H3K4me3 during oogenesis and early embryogenesis attracted much attention (24). Apart from the transcriptional regulatory functions, H3K4me3 is required for meiotic progression (12) and can serve as a mark of active meiotic recombination initiation sites (25), suggesting transcription-independent roles of H3K4me3. In this study, we characterized H3K4me3 deposited on chromosomes of mouse MII oocytes, whose functions have not been assessed in the previous report since the lack of H3K4me3 in immature oocytes led to meiotic arrest before reaching the MII stage (12). We directly removed H3K4me3 from MII oocytes by overexpressing an H3K4 demethylase, enabling us to assess the functions of H3K4me3 in a transcriptionally inactive environment. Reduced H3K4me3 levels in oocyte genomes resulted in the abnormal structure of the meiotic apparatus (Fig. 3*B*) and impaired preimplantation development after IVF (Fig. 3*C*). Furthermore, we observed decreased H3K4me3 levels in oocytes from aged mice (Fig. 3*F*), in good agreement with the previous report (19), implying that the maintenance of H3K4me3 in meiotic chromosomes may secure normal development. Our results support the notion that H3K4me3 has a transcription-independent role during meiosis. Further investigations will clarify the H3K4me3-mediated chromosomal functions for meiotic division.

One of the interesting observations in this study is that H3K4me3 is preferentially deposited onto the chromosomes, which are closely located to the cortex. Chromosome labeling experiments revealed that chromosome X tends to be located on the cortex side. It has been recently shown that larger chromosomes are preferentially at the outer region of the metaphase plate in MI oocytes (15). Therefore, it is reasonable that the X chromosome often appeared near the cortex in MII oocytes. However, the asymmetric histone modifications on MII chromosomes have not been previously reported, although asymmetry in the meiotic spindle can be generated by CDC42 signaling from the cell cortex (26). Possible explanations for this phenomenon are 1) histone H3K4 methylases are localized near the cortex side of the meiotic apparatus or 2) chromosomes that are highly methylated in H3K4 are preferentially moved to the cortex side. The inhibition experiment of the actin cap formation by the Arp2/3 inhibitor treatment resulted in the dislocation of the meiotic apparatus, leading to evenly distributed H3K4me3 on oocyte chromosomes. Taken together, our obtained results favor the former explanation. It would be intriguing to examine the localization of histone methyltransferases in MII oocytes. We have not tested the functional significance of cortically localized H3K4me3 on MII chromosomes since it is technically challenging to specifically lower H3K4me3 at the cortex side. It might be possible to perform such functional experiments by finding and perturbing specifically localized enzymes for oocyte H3K4me3, if any. Our study thus shows the unique localization pattern of H3K4me3 on the MII oocyte genome, and the importance of meiotically accumulated H3K4me3 for the following development.

## Experimental procedures

Full experimental procedures can be found in the Supporting Information.

### Animals

Mice (DBA/2, ICR strains, and B6D2F1 [C57BL/6J × DBA/2N)] at 8 to 12 weeks of age were purchased from CLEA Japan, Japan SLC, or Kiwa Laboratory Animals, and maintained in light-controlled, air-conditioned rooms. Mice at 48 weeks of age were regarded as aged mice. DBA/2 or ICR mice were used for IVF as reported previously (27). This study was carried out in strict accordance with the recommendations in the Guidelines of Kindai University and RIKEN for the Care and Use of Laboratory Animals. Experimental protocols were approved by the Committee on the Ethics of Animal Experiments of Kindai University (Permit Number: KABT-31-003) and the Institutional Animal Care and Use Committee at RIKEN Kobe Branch. All mice were sacrificed by cervical dislocation, and all efforts were made to minimize suffering and to reduce the number of animals used in the present study.

## Data availability

All the image data generated in this study are available from the corresponding author (miyamoto.kei.823@m.kyushu-u.ac.jp) upon reasonable request.

## Supporting information

This article contains supporting information (28, 29, 30).

## Conflict of interest

The authors declare that they have no conflicts of interest with the contents of this article.
