# Supplementary material for: Characterization of H3K4me3 in mouse oocytes at the metaphase II stage[image]
Source: J Biol Chem. 2025 May 29;301(7):110308. doi: 10.1016/j.jbc.2025.110308 (PMC12240069; doi:10.1016/j.jbc.2025.110308)
Supplement: Supporting information [file mmc1.pdf]

Supporting Information

Characterization of H3K4me3 in mouse oocytes at the metaphase II stage

Atsushi Takasu[1], Toshiaki Hino[2], Osamu Takenouchi[3], Yasuki Miyagawa[4], Zhihua Liang[1], Shota Tanaka[1], Tomoya Mimura[1], Chisato Ida[1], Yuki Matsuo[1], Yuna Lee[1], Haruka Ikegami[1], Miho Ohsugi[5], Shogo Matoba[6], Atsuo Ogura[6], Kazuo Yamagata[1], Kazuya Matsumoto[1], Tomoya S Kitajima[3], Kei Miyamoto[1, 4, 7 *]

[1]Faculty of Biology-Oriented Science and Technology, Kindai University, Wakayama 649-6493, Japan.

[2]Department of Biological Sciences, Asahikawa Medical University, Asahikawa, Japan.

[3]Laboratory for Chromosome Segregation, RIKEN Center for Biosystems Dynamics Research (BDR), Kobe, Japan.

[4]Laboratory of Animal Reproductive Physiology, Faculty of Agriculture, Kyushu University, Fukuoka 819-0395, Japan.

[5]Department of Biological Sciences, Graduate School of Science, The University of Tokyo, Hongo 7-3-1, Bunkyo-ku, Tokyo, 113-0033, Japan.

[6]Bioresource Engineering Division, RIKEN Bioresource Research Center, Tsukuba, Ibaraki 305-0074, Japan.

[7]Environmental Control Center for Experimental Biology, Kyushu University, Fukuoka 819-0395, Japan.

[*]Corresponding author

E-mail: miyamoto.kei.823@m.kyushu-u.ac.jp

Telephone: +81-92-802-4589 (Kei Miyamoto)

Supplemental Experimental Procedures

Mouse oocyte collection and culture

Female mice (>8 weeks of age) were injected with 10 IU of pregnant mare serum gonadotropin (PMSG) and 48 h later, human chorionic gonadotropin (hCG). Cumulus-oocyte complexes were recovered into pre-equilibrated human tubal fluid (HTF) medium (ARK Resource) and then denuded by 0.1% hyaluronidase in HTF medium at 37°C until almost complete dispersion of cumulus cells for the following injection experiments. The denuded oocytes were cultured in M16 medium (M7292; Sigma-Aldrich) at 37°C in air containing 5% CO_2.

Micro-manipulation of oocytes

Cumulus-free MII oocytes were washed and kept in drops of M2 medium, when taken out of the incubator. The injection was performed in CZB-HEPES medium. mRNAs were injected using a piezo manipulator (Prime Tech). The final concentrations of injected mRNA are as follows; 850 ng/μl *Kdm5b-CD*^{WT}, 850 ng/μl *Kdm5b-CD*^{i}, 850 ng/μl full length *Kdm5b* and 250 ng/μl *GFP*. After injection, embryos were incubated in M16 medium for 6 hours at 37°C in air containing 5% CO_2, allowing translation of the injected mRNA.

IVF and embryo culture

The collection of spermatozoa was performed as described in previous studies (27, 28). Briefly, spermatozoa were collected from the cauda epididymis of fertile male mice (>12 weeks of age). The sperm suspension was incubated in HTF medium for 1.5 hours to allow for capacitation at 37°C under 5% CO_2 in air. Oocytes were prepared as described above. The mRNA-injected oocytes were cultured in M16 medium for 6 hours before *in vitro* fertilization. A small hole of zona pellucida was opened by the XYClone laser system (Hamilton Thorne) for sperm to gain access to an oocyte. The sperm suspension was added to the oocyte cultures and

morphologically normal zygotes were collected 2 hours post insemination (hpi). The zygotes were cultured in potassium simplex optimized medium KSOMaa (ARK Resource) at 37°C under 5% CO_2 in air.

CK666 treatment

Stock solution of CK666 was prepared at a concentration of 50 mM in DMSO, and diluted in M16 medium to a final concentration of 250 μM for inhibiting Arp2/3 dynamics (21). MII oocytes were then cultured in M16 medium containing CK666 for 3 hours and used for immunofluorescence analyses. The control group was cultured in M16 medium containing DMSO (vehicle control).

Live imaging of in vitro matured oocytes for CRISPR-Sirius-based labeling of chromosomes

Oocytes at the germinal vesicle stage were isolated from the ovaries 48 hours after injection of CARD HyperOva (Kyudo) into female mice. The fully grown oocytes were released and collected in M2 medium containing 3-isobutyl-1-metyl-xanthine (IBMX, 15879, Sigma). The mixture of mRNA, sgRNA and purified dCas9 protein (Fig. 2D) was introduced by microinjection and cultured for at least 3.5 hours at 37°C. For labeling the sequences in chromosomes 8 and X, 0.16 pg of sgRNA was injected. The *in vitro* transcribed mRNAs (1.8 pg *H2B-SNAP*, 0.9 pg *Major-satellite-TALE-tdTomato*, 2.7 pg *PCP-3mClover*, and 2.7 pg *MCP-3tagBFP*) were introduced through microinjection. Purified proteins were injected: 5.9 pg dCas9 (1081067, Integrated DNA Technologies). All components were mixed in RNase-free water before injection. Then, the injected oocytes were cultured in a medium containing SNAP-Cell 647-SiR (S9102S, NEB) with IBMX for 30 min at 37°C. The oocytes were released from the SNAP-Cell 647-SiR and IBMX by washing to induce meiotic resumption.

Live cell imaging was performed as previously described (29, 30). In brief, a Zeiss LSM780 confocal microscope equipped with a GaAsP detector and a 40× C-Apochromat 1.2 NA

water immersion objective lens was controlled with AutoFocuscreen and MyPiC. For chromosome imaging, 19 or 31 confocal z sections (every 1.5 μm, 1.0 μm) of 512×512 pixel xy images were acquired every 15 min. All imaging was performed for at least 16 hours after induction of meiotic resumption.

Immunofluorescence analysis

Embryos were treated with 4% PFA containing 0.2% Triton X-100/PBS, at RT for 15 min, and were washed with 1 mg/ml PVP/PBS three times. Alternatively, embryos were fixed with 4% PFA and then permeabilized with 0.5% Triton X-100/PBS for 15 min for Figs 1, G and H, and S1C. For staining of 5-mC and 5-hmC, the permeabilized oocytes were washed three times with PBS, and then incubated in 4N HCl for 10 min, followed by the addition of 100 mM Tris-HCl (pH 8.5) for neutralization. Blocking was performed in 3% BSA/PBS with 30 min at RT, then embryos were incubated with primary antibodies diluted in 3% BSA/PBS with (1:500; Anti-histone H3K4me3 antibody (pAb) [39159; Active Motif], 1:500; Anti-Histone H3K4me3 antibody [ab8580; abcam], 1:500; Anti-Histone H3K36me3 antibody [61022; Active Motif], 1:200; Anti-α-Tubulin Antibody [#2144; Cell Signaling], 1:500; Anti-Histone H3K9me antibody [ab8898; abcam], 1:500; AbFlex® Histone H3.3 antibody [91191; Active Motif], 1:1000; Anti-5-Methylcytosine (5-mC) Mouse mAb (162 33 D3) [NA81; Merck] and 1:1,000; Anti-5-Hydroxymethylcytosine (5-hmC) antibody [39769; Active Motif] at 4°C overnight. After three times washes by 1% BSA/PBS, samples were further incubated in the dark with Alexa Fluor 488-labeled goat anti-mouse IgG antibody (1:2,000; A11001; Thermo Fisher Scientific), Alexa Fluor 488-labeled goat anti-rabbit IgG antibody (1:2,000; A11008; Thermo Fisher Scientific), Alexa Fluor 594-labeled donkey anti-mouse IgG antibody (1:2,000; A21203; Thermo Fisher Scientific) or Alexa Fluor 594-labeled donkey anti-rabbit IgG antibody (1:2,000; A21207; Thermo Fisher Scientific) at RT for 1 h. The samples were washed with 1% BSA/PBS three times and then mounted on slides using VECTASHIELD Mounting Medium containing DAPI. The fluorescence

signals were observed using a Zeiss LSM800 microscope, equipped with a laser module (405/488/561/640 nm) and GaAsP detector, using the same contrast, brightness, and exposure settings within the same experiments. Z-slice thickness was determined by using the optimal interval function in the ZEN software.

For staining with phalloidin, oocytes treated with 250 μM CK666 or DMSO were fixed with 4% PFA containing 0.2% Triton X-100/PBS, at RT for 15 min, followed by three times washes with 3% BSA/PBS. Those permeabilized oocytes were stained with 100 nM Acti-stain™ 555 Phalloidin (PHDH1; Cytoskeleton). After three times washes with 1% BSA/PBS, the samples were then mounted on slides using VECTASHIELD Mounting Medium containing DAPI. The fluorescence signals were observed using a Zeiss LSM800 microscope, as described above.

Immunostaining and multicolor FISH of MII oocytes using the same chromosome slides

Preparation of chromosome slide of MII oocytes and multicolor FISH analysis of MII oocytes was performed as described previously (14). The oocytes were treated with 0.5% actinase E (Kaken Pharmaceutical Co.) to dissolve the zona pellucida and incubated in a hypotonic solution (1:1 mixture of 1.2% sodium citrate and 18% fetal bovine serum) for 10 min. The oocytes were fixed with fixative I (5:1:4 mixture of methanol, acetic acid and distilled water) for 5 to 10 min, mounted gently on a slide with a small quantity of fixative I, and covered with a gentle flow of fixative II (3:1 mixture of methanol and acetic acid). The slide was placed into a Coplin jar containing fixative II and left for 1-2 min. Finally, the slide was dipped in fixative III (3:3:1 mixture of methanol, acetic acid and distilled water) for 1 min and air dried at 22–24°C under 50–60% humidity.

The chromosome slide was permeabilized with 0.1% Triton X/PBS for 10 min, blocked with 10% BSA/PBS, and incubated with anti H3K4me3 or H3.3 antibody (1:500) overnight at 4°C in blocking solution. After incubation with AlexaFlour 488- or 594-conjugated secondary antibody (1:500) at RT for 1 h, the slide was covered by a coverslip with VECTASHIELD

mounting medium containing DAPI and observed under a fluorescent microscope (BX51; Olympus, Tokyo, Japan) equipped with a high-sensitivity digital camera (α7s; Sony, Tokyo, Japan).

After capturing the fluorescent images, the chromosome slide was washed in PBS for 15-20 min and dehydrated in 70%, 95%, and 100% ethanol for 1 min at RT each. The slide was air dried and incubated in a solution of 2× sodium chloride–sodium citrate (SSC) for 30 min at 70°C. The slide was denatured in 0.07 M NaOH for 1 min at 4°C and washed in 0.1× SSC and in 2× SSC for 1 min at 4°C each. Subsequently, the slide underwent a second round of sequential dehydration in 70%, 95%, and 100% ethanol for 1 min at RT and was air dried. Prior to hybridization, a multicolor FISH probe (21xMouse; MetaSystems Probes, Altlußheim, Germany) was denatured for 5 min at 75°C and incubated for 30 min at 37°C. The probe was applied to the slide, sealed with a cover slip using rubber cement, and incubated in a moist chamber for 48 h at 37°C. After hybridization, the slide was washed in 0.4× SSC for 2 min at 72°C and 2× SSC with 0.05% Tween20 for 30 s at RT. The slide was rinsed in distilled water, air dried, and mounted with DAPI/Antifade (MetaSystems Probes). The fluorescent images of the chromosome spreads were captured using a fluorescence microscope (BX51; Olympus) equipped with a high-sensitivity digital camera (α7s; Sony). The images were processed using ChromaWizard software for karyotyping.

Image analysis

Images were analyzed using the ZEN software, Imaris (Bitplane), Image J, and Python with the OpenCV library (version 4.11.0). For the quantification of target signals in MII oocyte, all focal planes were merged by maximum intensity projection (MIP). Processing of MIP images was carried out using a Python script by incorporating the OpenCV and raw image data in CZI format. The DAPI channel was extracted from each image and converted to an 8-bit grayscale image. To enhance contrast and reduce noise, a Gaussian blur with a kernel size of 5×5 (cv2.GaussianBlur)

was applied, followed by binarization using thresholding (cv2.threshold). Clearly defined chromosomal structures stained with DAPI were extracted by contour detection using cv2.findContours. The area of each contour was calculated with cv2.contourArea, and regions with an area less than 500 px^2 were excluded as non-specific background or fragmented structures. Remaining chromosomal regions larger than 500 px^2 were identified using cv2.boundingRect and used for subsequent analyses. The centroid coordinates of the detected chromosomal regions (ROI centers) were calculated. The signal intensities of the ROI were measured. A line was defined from the ROI center toward the center of the oocyte, and perpendicular to this line, pixels were reorganized and divided into a specified number of bins to calculate the average DAPI and target signal intensities in each bin.

In Figs. 1D, 1F, 1G, 1H, S1A, S1C, S1D, S1E, 3A, 3E, and S3B, the number of bins was set to 2, resulting in a division of the chromosomal region into cortical and central sides for comparison. In Figs. 1C, 1F, S1E, and 3D, the chromosomal region was divided into 10 bins from the cortical side toward the center to generate line plots. In Fig. S3A, 3F, 4C, and 4D, the number of bins was set to 1, and the average signal intensity over the entire chromosomal region was compared.

In Figs. 3B, S3C, and S3F, the collected MII oocytes were fixed and stained with tubulin and DAPI as described above, and the lengths of the meiotic apparatus were measured.

In Figs. 4E and 4F, regardless of cortical or central position, the chromosomal area was radially divided into six equal-angle sectors (60° each) (Fig. S4). A sector with the highest signal intensity within the chromosomal area was designated as SECTOR 1. Other sectors were positioned accordingly; the two sectors adjacent to SECTOR 1 were assigned as SECTORs 2 and 3, and the remaining ones were sequentially assigned as SECTORs 4 to 6 (see Fig. S4). Finally, the average signal intensities of SECTORs 1–3 (High group) and SECTORs 4–6 (Low group), both normalized by DAPI signal, were compared.

For Figs. 2F and S2A, Imaris was used to construct 3D images of mouse oocyte

chromosomes. To measure chromosome volumes, 3D surface rendering with the signal of H2B-SNAP was performed using Imaris. Histone H2B volumes were calculated by the Imaris-equipped algorithm. The target chromosomes were identified based on the fluorescent spots of CRISPR-Sirius (Fig. 2E). To determine the coordinates of the nearest cell membrane, the distance from the central coordinates of H2B signals (shown as G in Fig. 2F) to the cell membrane (shown as M in Fig. 2F) was calculated (designated as R_0). The distance from each chromosome to the cell membrane (R_X and R_8 in Fig. 2F) was then normalized by the distance from the central H2B coordinates to the neighboring cell membrane coordinates (R_0).

mRNA and sgRNA production

The catalytic domain of mouse *Kdm5b* (accession number NM_152895, aa1-770) and its catalytic inactive mutant (H499A; aa1-770) were cloned into the pCS2 vector with a C-terminal NLS-tag (16). The full length of *Kdm5b* was cloned into the pcDNA3.1 vector. mRNAs were then prepared from pCS2 or pcDNA3.1 vectors using mMESSAGE mMACHINE SP6 or T7 Transcription Kit (Thermo Fisher Scientific, AM1340 or AM1344) or MEGAscript T7 Transcription Kit (Thermo Fisher Scientific, AM1334). Briefly, to produce linearized vectors, approximately 5 mg plasmids were digested overnight, with appropriate restriction enzymes. In the case of pCS2 vectors, the produced mRNAs were subjected to adding polyA tails (Thermo Fisher Scientific, AM1350), while pcDNA3.1 vectors that already possessed polyA tails were transcribed from the T7 promoter. Produced mRNAs were purified using RNeasy Mini Kit (QIAGEN, 74106).

sgRNAs and mRNA for chromosome labeling experiments were transcribed from the pGEMHE vector using mMESSAGE mMACHINE T7 Kit (AM1344, Ambion). For a template of sgRNA synthesis, a T7-promoter-conjugated DNA fragment was amplified by PCR. The templates were purified on a 1% agarose gel. H2B-SNAP was obtained from New England Biolabs (N9186).

Statistics test

All of the statistical methods are described in the figure legends. The chi-square test was used for Figs. 3C and S3D to evaluate the differences in developmental rates. Following the F-test, an appropriate T-test was used for Figs. 1, D and F-H, S1, A and C-E, 2F, 3, B and E-F, S3, A-C and F, and 4, C and D (unpaired, 2-tailed) and Figs. 4, E and F (paired, 2-tailed). For other analyses to evaluate the differences between groups, one-way ANOVA followed by a Tukey's LSD test was used. Differences were considered significant at P-values < 0.05.

Supplemental Figures

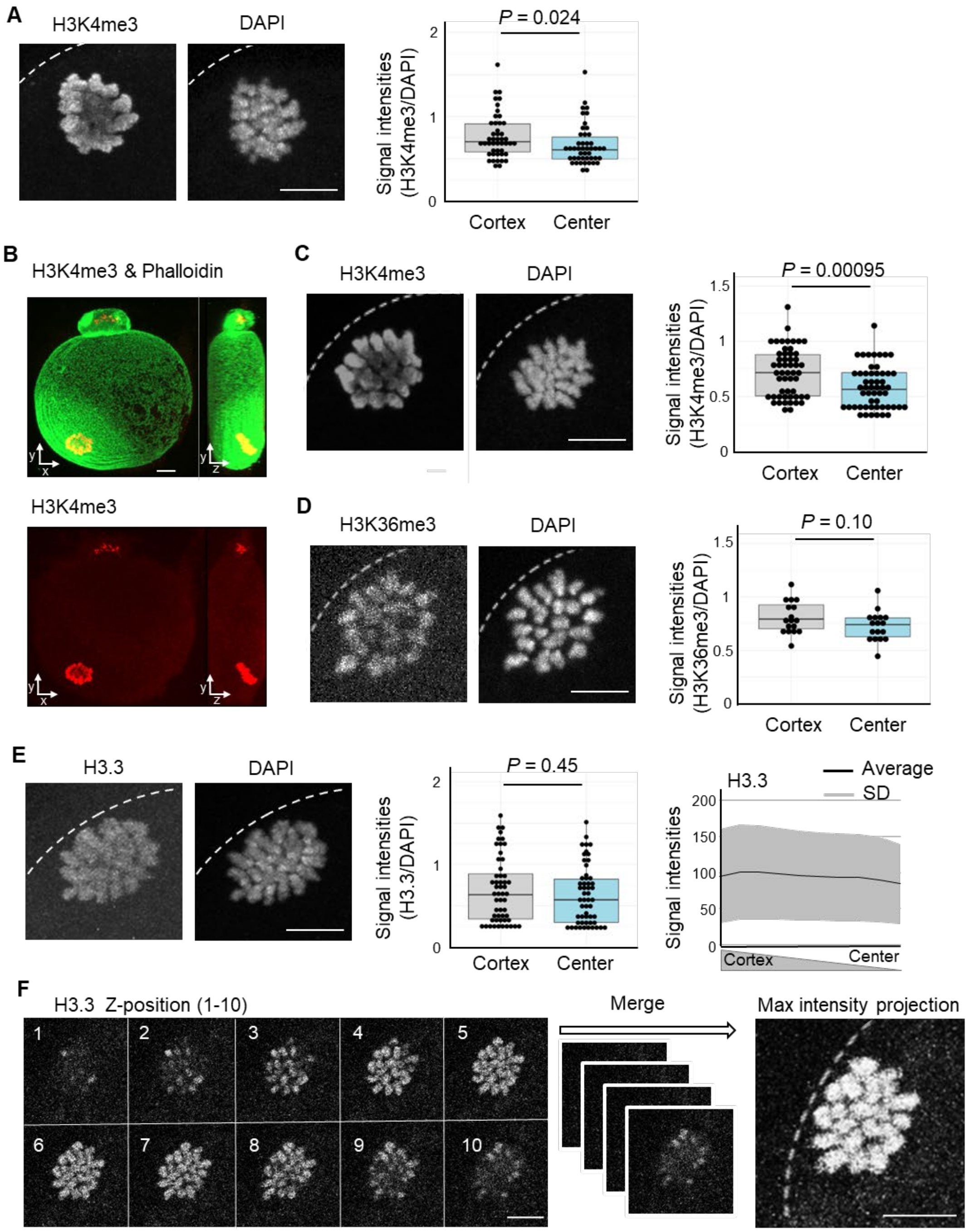

Figure S1. The accumulation of H3K4me3 in MII oocyte chromosomes on the cortical side. (A) Immunostaining of H3K4me3 using MII oocytes with the different antibody from Fig. 1A and its quantification, as performed in Figs. 1, B-D. N = 10 independent experiments, n = 85 oocytes. P value was determined by t-test. Scale bar, 10 µm.

(B) Co-immunostaining of the cortical actin and H3K4me3 (red) using MII oocytes. The cortically accumulated polymerized actin was visualized by phalloidin staining (green). Z or X planes were merged by maximum intensity projection. N = 2 independent experiments, n = 18 oocytes.

(C) Immunostaining of H3K4me3 using MII oocytes, prepared by a different immunostaining protocol, and its quantification. N = 3 independent experiments, n = 53 oocytes. P value was determined by t-test. Scale bar, 10 μm.

(D) Immunostaining of H3K36me3 using MII oocytes and its quantification. N = 2 independent experiments, n = 17 oocytes. P value was determined by t-test. Scale bar, 10 μm.

(E) Immunostaining of H3.3 using MII oocytes and its quantification. SD represents standard deviation. N = 5 independent experiments, n = 53 oocytes. P value was determined by t-test. Scale bar, 10 μm.

(F) Immunostaining images of H3.3 across z-stacks using an MII oocyte. All focal planes were merged by maximum intensity projection.

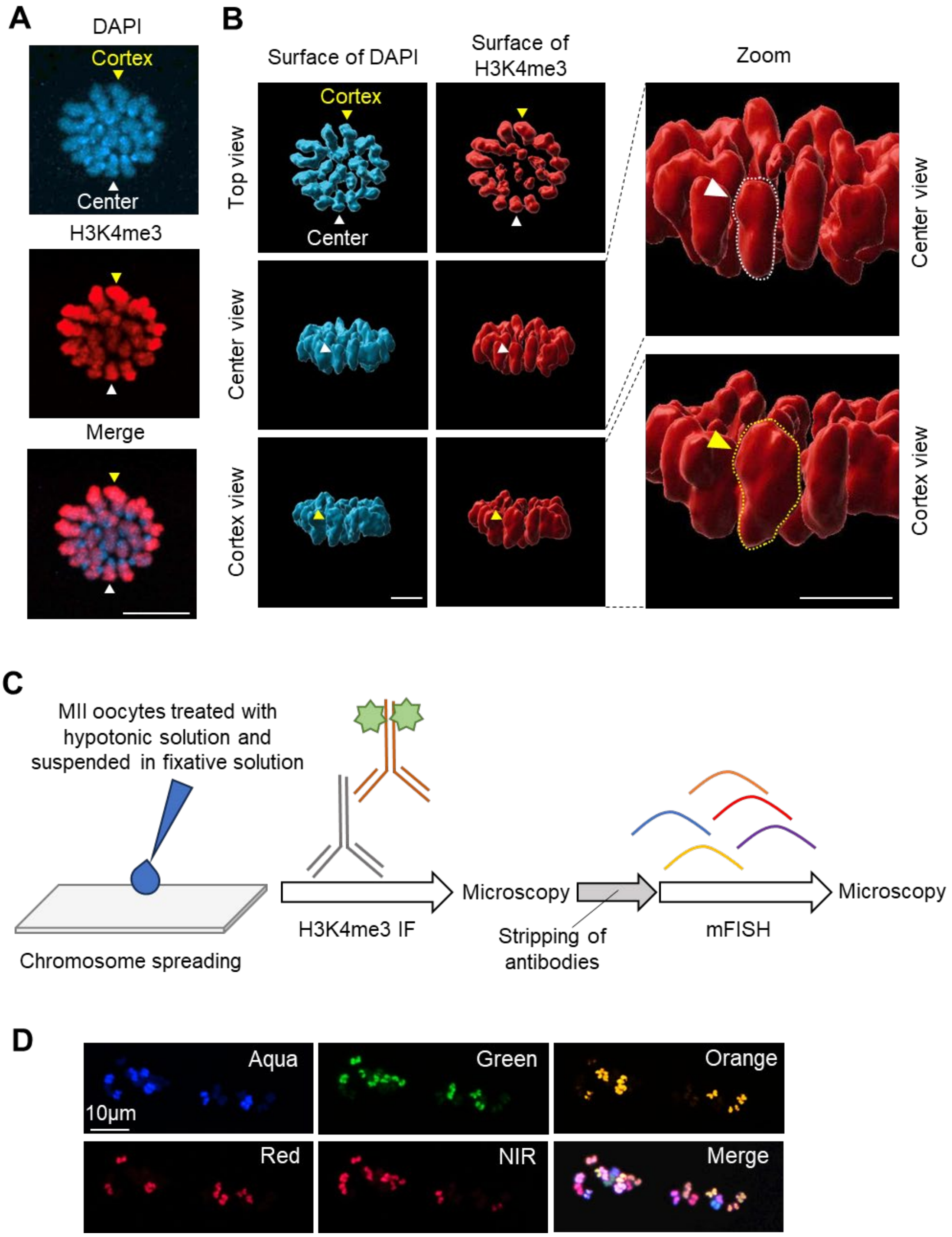

Figure S2. 3D analysis of H3K4me3 immunostaining on MII oocyte chromosomes.

(A) A representative image of immunostaining of H3K4me3 using a mouse MII oocyte. DNA was stained with DAPI. Merge: merged photo. The cortex and center sides are indicated by arrowheads. Scale bar, 10 µm.

(B) 3D images were constructed by Imaris using confocal images of mouse MII oocyte chromosomes stained with H3K4me3 (red) and DAPI (blue). Corresponding chromosomes stained with H3K4me3 and DAPI are shown in (A). Scale bar, 5 μm.

(C) A schematic diagram showing the process of immunofluorescence analyses, followed by mFISH using the same oocyte samples. This method allows the comparison of H3K4me3 signals among different chromosomes as in Fig. 2A-C.

(D) Representative images of multicolor FISH. Chromosomes were stained with 5 different dyes (Aqua, Green, Orange, Red, NIR) to identify each chromosome number. The merged photo at the bottom, right panel corresponds to the merged image shown in Fig. 2A.

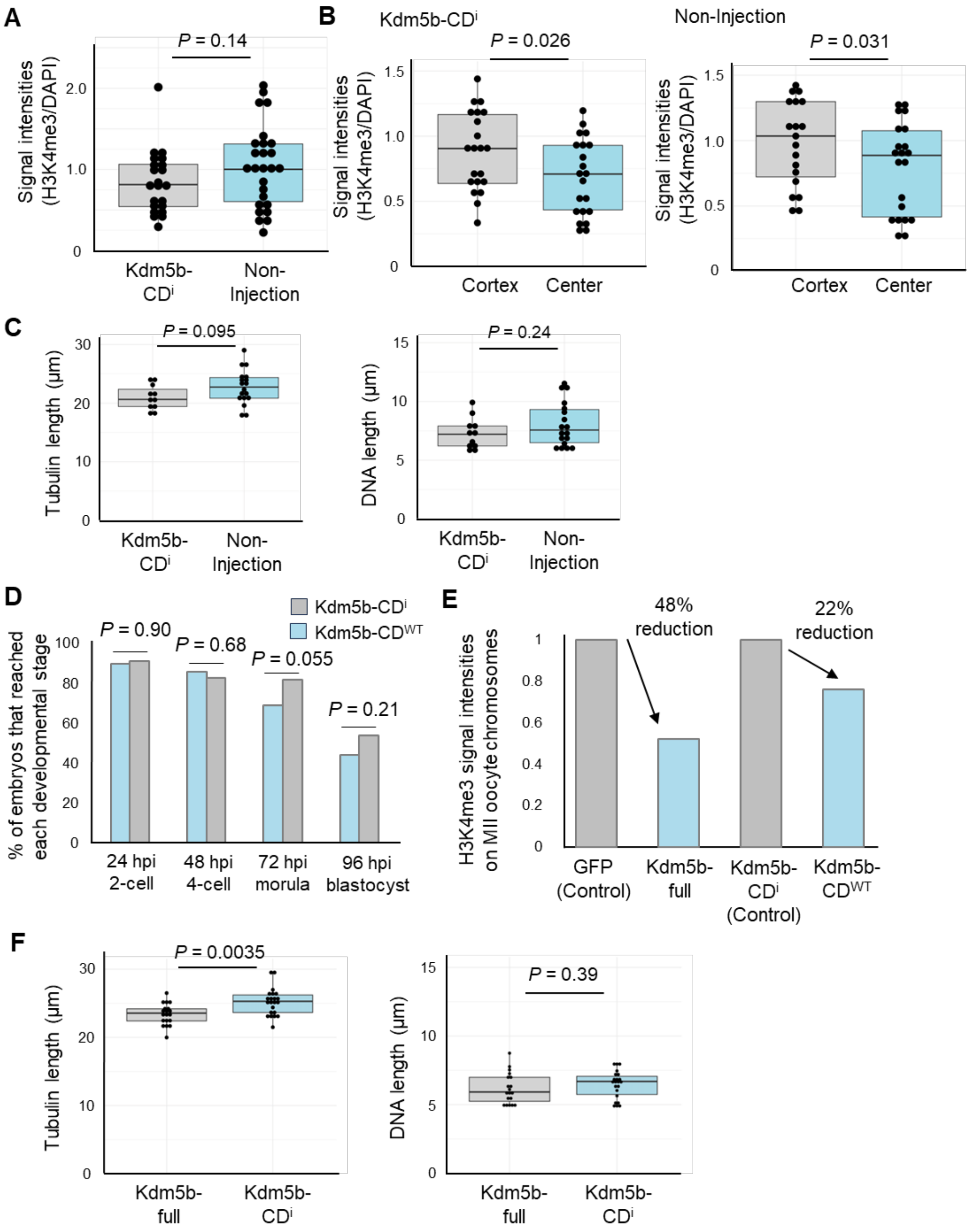

Figure S3. Preimplantation development and phenotypic changes of the meiotic apparatus after lowering H3K4me3 levels in MII oocyte chromosomes.

(A) H3K4me3 signal intensities on MII oocyte chromosomes were not changed after the overexpression of *Kdm5b-CD*^{i} in MII oocytes, as compared to non-injection control oocytes. Box plots show the quantification of H3K4me3/DAPI signal intensities. N= 3 independent

experiments, n = 20 (*Kdm5b-CD^i^*) and 21 (non-injection). P value was determined by t-test.

(B) Cortically localized H3K4me3 signals were maintained after overexpression of *Kdm5b-CD*^{i} mRNA. As a control, non-injection control was measured. N= 3 independent experiments, n = 20 (*Kdm5b-CD*^{i}) and 21 (non-injection). P values were determined by t-test.

(C) Tubulin and DNA sizes of MII oocyte chromosomes overexpressing *Kdm5b-CD*^{i} mRNA, as compared to non-injection control oocytes. Immunostaining of tubulin in mouse MII oocytes was performed, and the length of tubulin and DNA was measured. Box plots show the quantification of each parameter. N= 3 independent experiments, n = 11 (*Kdm5b-CD*^{i}) and 18 (non-injection). P values were determined by t-test.

(D) Preimplantation development after IVF of *Kdm5b-CD*^{WT}-overexpressed oocytes. Development to each developmental stage was observed every 24 hours post-insemination (hpi). As a control *Kdm5b-CD*^{i} mRNA was injected to oocytes. Statistical significances were calculated by the chi-square test.

(E) The overexpression of full-length Kdm5b efficiently reduced H3K4me3 levels in MII oocyte chromosomes when compared with that of Kdm5b-CD^{WT}. Signal intensities of H3K4me3 in control oocytes were set as 1. N= 2 independent experiments, n = 11 (full *Kdm5b* vs control [EGFP mRNA-injected oocytes]) and 20 (*Kdm5b-CD*^{WT} vs control [*Kdm5b-CD*^{i} mRNA-injected oocytes]).

(F) Tubulin and DNA sizes of MII oocyte chromosomes overexpressing full length *Kdm5b* mRNA, as compared to those overexpressing *Kdm5b-CD*^{i}. Immunostaining of tubulin in mouse MII oocytes was performed, and the lengths of tubulin and DNA were measured. Box plots show the quantification of each parameter. N= 3 independent experiments, n = 18 (full *Kdm5b*) and 11 (*Kdm5b-CD*^{i}). P values were determined by t-test.

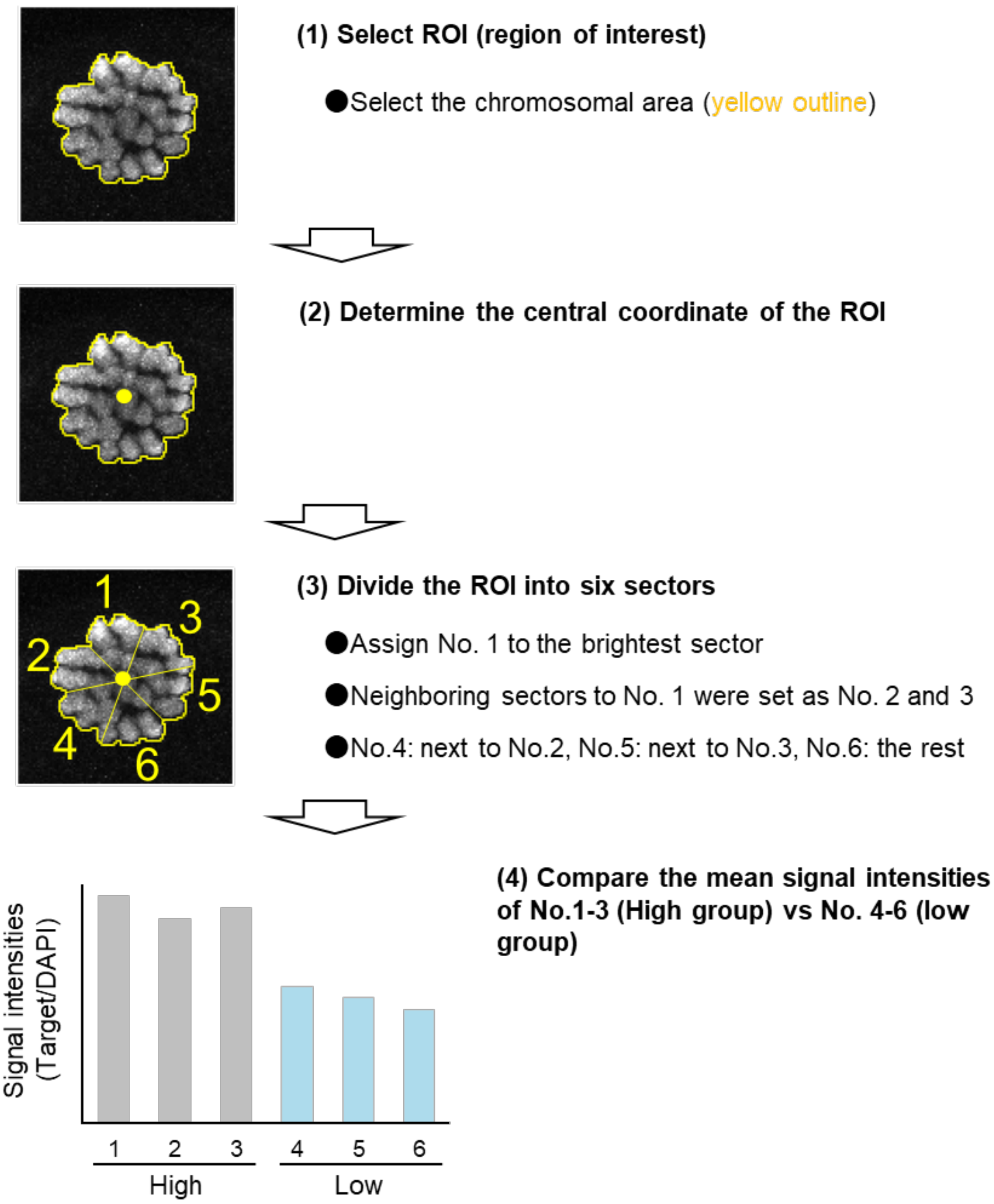

Figure S4. A schematic diagram shows a quantification method of signal intensity distribution on MII chromosomes.

Supplemental Video Legend

Video S1. 3D view of mouse MII oocyte chromosomes, constructed by Imaris using confocal images of mouse MII oocyte chromosomes stained with H3K4me3 (red) and DAPI (blue). Corresponding chromosomes stained with H3K4me3 and DAPI are shown in Fig. S2A.

Supplemental References

27. Okuno, T., Li, W. Y., Hatano, Y., Takasu, A., Sakamoto, Y., Yamamoto, M., Ikeda, Z., Shindo, T., Plessner, M., Morita, K., Matsumoto, K., Yamagata, K., Grosse, R., and Miyamoto, K. (2020) Zygotic Nuclear F-Actin Safeguards Embryonic Development. *Cell Reports*. **31**, 107824
28. Ihashi, S., Hamanaka, M., Kaji, M., Mori, R., Nishizaki, S., Mori, M., Imasato, Y., Inoue, K., Matoba, S., Ogonuki, N., Takasu, A., Nakamura, M., Matsumoto, K., Anzai, M., Ogura, A., Ikawa, M., and Miyamoto, K. (2023) Incomplete activation of Alyref and Gabpb1 leads to preimplantation arrest in cloned mouse embryos. *Life Science Alliance*. 10.26508/lsa.202302296
29. Sakakibara, Y., Hashimoto, S., Nakaoka, Y., Kouznetsova, A., Höög, C., and Kitajima, T. S. (2015) Bivalent separation into univalents precedes age-related meiosis I errors in oocytes. *Nat Commun*. **6**, 7550
30. Kitajima, T. S., Ohsugi, M., and Ellenberg, J. (2011) Complete Kinetochore Tracking Reveals Error-Prone Homologous Chromosome Biorientation in Mammalian Oocytes. *Cell*. **146**, 568–581
